# Fabrication of biocompatible porous scaffolds based on hydroxyapatite/collagen/chitosan composite for restoration of defected maxillofacial mandible bone

**DOI:** 10.1007/s40204-019-0113-x

**Published:** 2019-05-29

**Authors:** Md Shaifur Rahman, Md Masud Rana, Lucas-Sebastian Spitzhorn, Naznin Akhtar, Md Zahid Hasan, Naiyyum Choudhury, Tanja Fehm, Jan T. Czernuszka, James Adjaye, Sikder M. Asaduzzaman

**Affiliations:** 1 Institute for Stem Cell Research and Regenerative Medicine, Medical Faculty, Heinrich-Heine-Universität Düsseldorf, 40225 Düsseldorf, Germany; 2 Institute of Tissue Banking and Biomaterial Research, Atomic Energy Research Establishment, 1349 Dhaka, Bangladesh; 3 School of Medicine, Geelong Waurn Ponds Campus, Deakin University, Waurn Ponds, Victoria 3217 Australia; 4 Bangladesh Atomic Energy Regulatory Authority, Dhaka, Bangladesh; 5 Department of Obstetrics and Gynaecology, Medical Faculty, Heinrich-Heine-Universität Düsseldorf, 40225 Düsseldorf, Germany; 6 Department of Materials, University of Oxford, Oxford, OX1 3PH UK

**Keywords:** Hydroxyapatite, Collagen, Chitosan, Scaffold, Biocompatibility, AF-MSCs, Mandible bone defect, Bone tissue engineering

## Abstract

Fabrication of scaffolds from biomaterials for restoration of defected mandible bone has attained increased attention due to limited accessibility of natural bone for grafting. Hydroxyapatite (Ha), collagen type 1 (Col1) and chitosan (Cs) are widely used biomaterials which could be fabricated as a scaffold to overcome the paucity of bone substitutes. Here, rabbit Col1, shrimp Cs and bovine Ha were extracted and characterized with respect to physicochemical properties. Following the biocompatibility, degradability and cytotoxicity tests for Ha, Col1 and Cs a hydroxyapatite/collagen/chitosan (Ha·Col1·Cs) scaffold was fabricated using thermally induced phase separation technique. This scaffold was cross-linked with (1) either glutaraldehyde (GTA), (2) de-hydrothermal treatment (DTH), (3) irradiation (IR) and (4) 2-hydroxyethyl methacrylate (HEMA), resulting in four independent types (Ha·Col1·Cs-GTA, Ha·Col1·Cs-IR, Ha·Col1·Cs-DTH and Ha·Col1·Cs-HEMA). The developed composite scaffolds were porous with 3D interconnected fiber microstructure. However, Ha·Col1·Cs-IR and Ha·Col1·Cs-GTA showed better hydrophilicity and biodegradability. All four scaffolds showed desirable blood biocompatibility without cytotoxicity for brine shrimp. In vitro studies in the presence of human amniotic fluid-derived mesenchymal stem cells revealed that Ha·Col1·Cs-IR and Ha·Col1·Cs-DHT scaffolds were non-cytotoxic and compatible for cell attachment, growth and mineralization. Further, grafting of Ha·Col1·Cs-IR and Ha·Col1·Cs-DHT was performed in a surgically created non-load-bearing rabbit maxillofacial mandible defect model. Histological and radiological observations indicated the restoration of defected bone. Ha·Col1·Cs-IR and Ha·Col1·Cs-DHT could be used as an alternative treatment in bone defects and may contribute to further development of scaffolds for bone tissue engineering.

**Electronic supplementary material:**

The online version of this article (10.1007/s40204-019-0113-x) contains supplementary material, which is available to authorized users.

## Introduction

Bone fractures remain a challenge in reconstructive and rehabilitation surgery. For instance, periodontal bone defects are very common in developing world and require a large amount of medical resources (Wang et al. 2016; Gaihre et al. 2017). Currently, implantable biomaterials such as demineralized bone granules, auto- and allografts are available and clinically used. However, these approaches involve several drawbacks such as post-operative pain, increased blood loss, and secondary surgical wounds (Kumar et al. 2016; Oryan et al. 2014). Allografting could overcome these limitations, but it is associated with the risk of infectious diseases, an insufficient number of donors and high costs (Greenwald et al. 2012). Recombinant growth factors, cell-based engineered bone substitutes and commercial scaffolds are sophisticated alternatives which are widely accepted in the developed world but are too expensive for patients in the low-income countries (Mao and Mooney 2015; Tollemar et al. 2016; Tong et al. 2016a, b). Recently, the interest in the development of scaffolds from naturally available and low-cost bioactive materials has increased significantly (Baino et al. 2015; Yi et al. 2016). These materials could improve bone function by providing a suitable microenvironment for tissue growth and regeneration (Yu et al. 2013; Polo-Corrales et al. 2014; Maisani et al. 2017).

Since hydroxyapatite (Ha) and collagen type 1 (Col1) are the major constituents of human bone, they are widely studied as promising materials for scaffold preparation. For example, the usefulness of porous nano-Ha/Col scaffolds for restoration of critical-size bone defect was reported (Wang et al. 2017). Beside commercial available synthetic Ha, many laboratories have extracted Ha from bio-waste such as bovine bones (Wua et al. 2016; Kim et al. 2014; Sofronia et al. 2014). Bioactive molecules such as Col1 and chitosan (Cs) are also used as components for bone composite engineering (Wang et al. 2016; Croisier and Jérôme 2013; Maji et al. 2016; Tong et al. 2016a, b) which can be extracted from animal skins (Kukhareva et al. 2010; Pacak et al. 2014) and brine shrimp, respectively (Khan et al. 2005; Maji et al. 2016). For instance, human mandible bone defects were restored by grafting of Col1 in combination with dental pulp progenitor cells (d’Aquino et al. 2009; Chamieh et al. 2016). Cs has reactive amine and hydroxyl groups which promote osteoblast growth and in vivo bone formation (Levengood and Zhang 2014) and has structural similarities with glycosaminoglycans, a major component of bone and cartilage (Nagahama et al. 2009; Gravel et al. 2006). In an advanced study, Cs and Cs-hydrogel were used as a bio-printable ink for bone tissue engineering (Demirtaş et al. 2017).

Ha, Col1 and Cs were described to have desired properties such as tissue compatibility, antibacterial activity, non-toxicity, non-immunogenicity, non-carcinogenicity and solubility (Pallela et al. 2012). These components can form a direct chemical bond with living cells/tissues and promote tissue growth, which makes them interesting for the use in orthopedic and dental applications (Rodríguez-Vázquez et al. 2015; Echazú et al. 2016). For example, the applicability of collagen-hydroxyapatite scaffolds for restoration of the mandible in the rabbit has been demonstrated (Zhang et al. 2013). Additionally, human adipose-derived MSCs seeded into a Col-Ha scaffold-promoted ectopic bone formation after implantation in the mouse (Calabrese et al. 2017). Cs in combination with silk-fibroin was observed to be biocompatible with osteogenic potentials when transplanted in a rabbit mandible defects model, including TGF-β1 supplementation (Tong et al. 2016a, b).

Ha, Col1 and Cs are usually animal originated and easy to obtain. So far, composites out of Ha, Cs and Col1 for non-load-bearing mandible bone restoration have not been widely studied. We fabricated Ha·Col1·Cs scaffolds from in-house rabbit skin (Col1), bovine bones (Ha) and prawn shell (Cs) and modified them using different cross-linking methods. The resulting scaffolds were characterized for cytotoxicity, biodegradability, in vivo biocompatibility and physical, chemical, and morphological properties.

## Materials and methods

### Fabrication procedures of Ha·Col1·Cs scaffolds

The in-house extraction methods of Ha, Col1 and Cs from naturally available sources have been described in Supplementary Materials and Methods. Scaffolds were fabricated according to a previous described thermally induced phase separation method with some modifications (Chen et al. 2010). In brief, 3 g Ha was weighed into a flask and deionized water was added. The mixture was stirred at room temperature (RT) for 5 h and was ultra-sonicated until the Ha powder was thoroughly dispersed. At the same time, 70 mL of collagen solution (5 mg/mL) was transferred into another flask and stirred at RT. Then, 1.67 g of chitosan was added slowly to the collagen solution and stirred at RT to form a chitosan–collagen mixture. After that, the Ha solution was added to the collagen–chitosan mixture and stirred for 2 h to disperse thoroughly. Afterwards, the mixture was transferred to a mold and pre-frozen at − 40 °C for 24 h, followed by freeze-drying at − 55 °C using a constant cooling freeze-drying protocol.

The resulting freeze-dried scaffold was modified using four different methods: chemical cross-linking with (1) HEMA and (2) GTA solution and physical crosslinking by (3) DHT and (4) IR. Cross-linkings were accomplished by immersing the freeze-dried scaffold in a cross-linker solution containing 2.5% HEMA or 2.5% GTA solution, respectively, for 4 h at RT. The scaffolds were washed with deionized water for 1 h. After that, scaffolds were frozen and lyophilized as described above. For the DHT method, lyophilized scaffolds were put under a vacuum at a temperature of 110 °C for 24 h. For IR, fabricated scaffolds were irradiated at 5–30 kGy using Co60γ sources. The resulting scaffolds were named according to the methods used for modification: Ha·Col1·Cs-GTA, Ha·Col1·Cs-IR, Ha·Col1·Cs-DTH and Ha·Col1·Cs-HEMA.

### X-ray diffraction (XRD) analysis

The phase and crystallinity of Ha nano-powder were evaluated using XRD (X’Pert PRO PW 3040, PANalytical, Netherlands). The parameter was CuKα radiation with a wavelength of 1.78896 Å and over a range of 2*θ* from 10° to 70° angle, step size 0.02/s with 40 kV voltages and 30 mA current. The XRD pattern was analyzed and compared with “X”pert high score and “X’Pert plus” software [“Xpert Highscore” File No. 01-086-0740 (ICDD 2005)] to identify the phase (Degen et al. 2014; Markvardsen et al. 2008).

### Fourier-transform infrared spectroscopy (FTIR) analysis

The stretching frequencies of Ha were examined by FTIR analysis (FTIR 8400S, Shimadzu spectrophotometer, Japan) in the range of 4000–400 cm^−1^. For Col1, samples were directly placed in the sample holder (IRprestige21, Shimadzu, Japan). FTIR spectra were recorded with FTIR 8400S Shimadzu Spectrophotometer in the range of 4000–700 cm^−1^, at a resolution 4 cm^−1^, number of scan: 20 times. For identifying organic, polymeric and inorganic materials within the four distinct Ha·Col1·Cs scaffolds, infrared light was used.

### Scanning electron microscopy (SEM) imaging

The morphology of Ha crystals as well as the pore structure morphology of the Ha·Col1·Cs scaffolds was obtained by SEM (JSM 6490LA, Jeol, Japan). The surface of the scaffolds was platinum coated to make it conductive and then the samples were placed inside the SEM chamber.

### Determination of porosity and density of Ha·Col1·Cs scaffold

The density and porosity of the fabricated scaffolds were measured by liquid displacement test (LDT) using ethanol as the liquid. A sample with a known weight (*W*) was immersed in a graduated cylinder in a known volume of ethanol (*V*_1_) for 5 min. The total volume of ethanol in the cylinder and ethanol impregnated scaffold was *V*_2_. The ethanol impregnated scaffold was removed from the cylinder and the residual ethanol volume was recorded (*V*_3_). For each scaffold, five independent measurements were done (*n* = 5). The density (*d*) and the porosity (*Є*) of the scaffolds are calculated using these formulas:

1 $$ d = {\raise0.7ex\hbox{$W$} \!\mathord{\left/ {\vphantom {W {\left( {V_{2} - V_{3} } \right)}}}\right.\kern-0pt} \!\lower0.7ex\hbox{${\left( {V_{2} - V_{3} } \right)}$}}, $$

2 $$ \varepsilon = {\raise0.7ex\hbox{${\left( {V_{1} - V_{3} } \right)}$} \!\mathord{\left/ {\vphantom {{\left( {V_{1} - V_{3} } \right)} {\left( {V_{2} - V_{3} } \right)}}}\right.\kern-0pt} \!\lower0.7ex\hbox{${\left( {V_{2} - V_{3} } \right)}$}}. $$

### Determination of swelling ability of Ha·Col1·Cs scaffold

The swelling ability was determined by the percentage of water absorption. Dry weight of the scaffold was denoted as Wd.

In brief, the scaffolds were immersed in PBS buffer solution with pH 7.4 at 37 °C for 2, 24, 48 and 72 h. Afterward, the scaffolds were taken out and their wet weight (*W*_w1_) was measured. In this case, we assessed the swelling ability of the scaffold structure employing its porous structure. In the second measurement, the same swollen samples were pressed between filter paper to remove the fluid remaining in the pores and then weighed (*W*_w2_). In this way, the intrinsic water absorbance ability of the scaffold material was assessed. For each scaffold, five independent measurements were done (*n* = 5). The swelling ratio of the scaffold was defined as the ratio of the weight increase (*W*_w_ − *W*_d_) to the initial weight (*W*_d_) according to following equation:

3 $$ {\text{Swelling ability}}\left( \% \right) = (W_{\text{W}} - W_{\text{d}} )/W_{\text{d}} \times 100, $$

where $W_{\text{W}}$ represents *W*_w1_ or *W*_w2_.

### Stability test for Ha·Col1·Cs scaffolds

Dried scaffolds were immersed into two different aqueous solutions (pH 4 and 7) at RT for intervals from 1 day up to 5 days. Scaffolds were then removed and dried for 48 h in a vacuum oven at 50 °C. A second weighing was conducted to determine the stabilities of the scaffolds by determining their weight loss. The stability of the scaffolds in the aqueous solution was calculated with the following equation:

4 $$ S = (W_{2} /W_{1} ) \times 100, $$

where *S* is the percentage of the weights of the scaffolds remaining after the test, *W*_1_ and *W*_2_ are, respectively, the weights of dried scaffolds before and after the test.

### Assessment of mechanical strength

Samples with a diameter of 15 mm and a height of 14 mm were prepared for mechanical strength testing. The test was carried out using a mechanical testing machine (Z.05, Zwick/Roell, Germany) at RT. The cross-head speed was set at 2 mm/min. The compressive modulus was calculated from the slope of the stress–strain curve in the linear region (strain from 2 to 5%). Each measurement was repeated five times and the average value was calculated.

### Biodegradability study of Ha·Col1·Cs scaffolds

To study the biodegradability, scaffolds were immersed in PBS-containing lysozyme (10,000 U/ml) at 37 °C for up to 21 days. At specific time points, the scaffolds were washed in deionized water to remove ions absorbed on the surface. Consecutively, the samples were lyophilized.

The degradation of the scaffold was calculated using following equation:

5 $$ {\text{Biodegradability}}\left( \% \right) = \frac{{W_{\text{o}} - W_{\text{t}} }}{{W_{\text{o}} }} \times 100, $$

where *W*_0_ is the initial and *W*_t_ is the dry weight of the scaffold.

### In vitro cytotoxicity and biocompatibility assay for Ha, Col1, Cs and Ha·Col1·Cs scaffolds

Cytotoxicity tests of the extracted Col1, Ha, Cs and scaffolds on brine shrimp (*Artemia salina*) were performed as described (Khan et al. 2012).

For in vitro blood biocompatibility assay, heparinized human blood was used. Ha, Col1 and Cs and Ha·Col1·Cs scaffolds powder were diluted with different ratios of blood. Blood sample diluted at the same ratios with deionized water and PBS served as controls. These mixtures were spread on glass slides after 2 h incubation at RT and observed under a light microscope.

### In vitro cell culture, attachment and growth in the presence of scaffold

Amniotic fluid-derived mesenchymal stem cells (AF-MSCs) were isolated and cultured in Chang C media containing 88% αMEM (Minimum Essential Medium Eagle Alpha Modification; Sigma) with 10% FBS, 1% GlutaMAX, 1% penicillin/streptomycin (all Gibco), 10% Chang B Basal Medium, and 2% Chang C supplement (Irvine Scientific) as described earlier (Spitzhorn et al. 2017). For observing the attachment, compatibility and growth of AF-MSCs in the presence of the Ha·Col1·Cs scaffolds, equal numbers of cells were seeded in 12-well plates with equal amounts scaffold nano-powder. Wells without any scaffold powder served as negative controls. Light microscopy images of the cells were taken on days 2, 5 and 10.

### In vitro degradability and mineralization study

To test the degradability of the scaffolds by AF-MSCs, we continued the cell attachment and growth assay for 21 days and the size-reduction of scaffold nano-powder particles was observed microscopically. After 21 days, upon degradation of the nano-powder, mineralized nodules were formed at the site of clustered AF-MSCs which were confirmed using alizarin red staining. The respective wells were washed three times with PBS and stained as described previously (Rahman et al. 2018).

### Histological and radiological analysis of in vivo grafted Ha·Col1·Cs scaffolds

The experimental model used in this study was a surgically created mandible critical-sized defect in rabbits (*Oryctolagus cuniculus*). Prior to the study, ethical approval was obtained from the institutional animal ethics committee of atomic energy research establishment. The surgery protocol was conducted in accordance with the regulations on animal welfare and complied with the guidelines.

During the experimental period, the rabbits were held in cages with pelleted food, hay and water at RT in a humidity controlled room. All animals were subjected to 12 h day/night cycles. Importantly, the animals were acclimatized for 15 days prior to surgery. Total 16 adult male rabbits (1.5–2.0 kg) were recruited for the study and assigned in 4 groups: mandibular defects were implanted with (1) Ha·Col1·Cs-IR, (2) Ha·Col1·Cs-DHT, and (3) human bone graft/chips as a positive control, or (4) empty defect as a negative control. Under aseptic conditions, the rabbits were first intravenously anesthetized with 2% pentobarbital sodium (30 mg/kg). The hair on the site of the mandible was then trimmed. Then a 20 mm longitudinal skin incision was made and subcutaneous tissues were separated gently along the upper edge of the rabbit mandible. An appropriate defect size of 15 mm height × 3 mm width × 2 mm depth was made, using an orthopedic hand drill machine with a drill bit size of 1.5 mm, under saline irrigation to avoid thermal necrosis. To make the scaffolds compliant and resilient, the scaffolds were soaked in blood that oozed out from the incision during surgery. The scaffold constructs were then implanted into the defect. Subsequently, the skin incision was then sutured with nylon, using horizontal mattress sutures. The surgical wound was cleaned with povidone iodine (5%) and dressed with nitro-furazone ointment. Animals were housed in individual pens for 7 days post-surgery to restrict activity during the initial stages of healing and then transferred to group pens for the remainder of the study. Analgesia and antibiotics were administered after surgery. Inj. Ceftriaxone 250 mg (TRIZON VET, ACME Laboratories Ltd, Bangladesh) was administered twice for 7 days Intra-muscular (I/M) and Inj. ketoprofen (K-Pain Vet, ACI Limited, Bangladesh) 0.5 mL was administered once daily (I/M). Sutures were removed on day 8. After 4 months, the experimental animals were killed, the skin was excised and the mandible bone samples of the scaffold-treated site were surgically collected for histological examinations. Excised bone fragments were fixed, dehydrated, and embedded in paraffin and cut into 5 mm sections with a microtome. Tissue sections were subjected to hematoxylin and eosin (H&E) staining and were viewed with a light microscope (Olympus BX51, Japan). X-ray radiological images were taken to observe in vivo bone formation at several time points during the progression of recovery.

## Results

### Fabrication of Ha·Col1·Cs scaffolds from hydroxyapatite, collagen, and chitosan

**Fig. 1:**
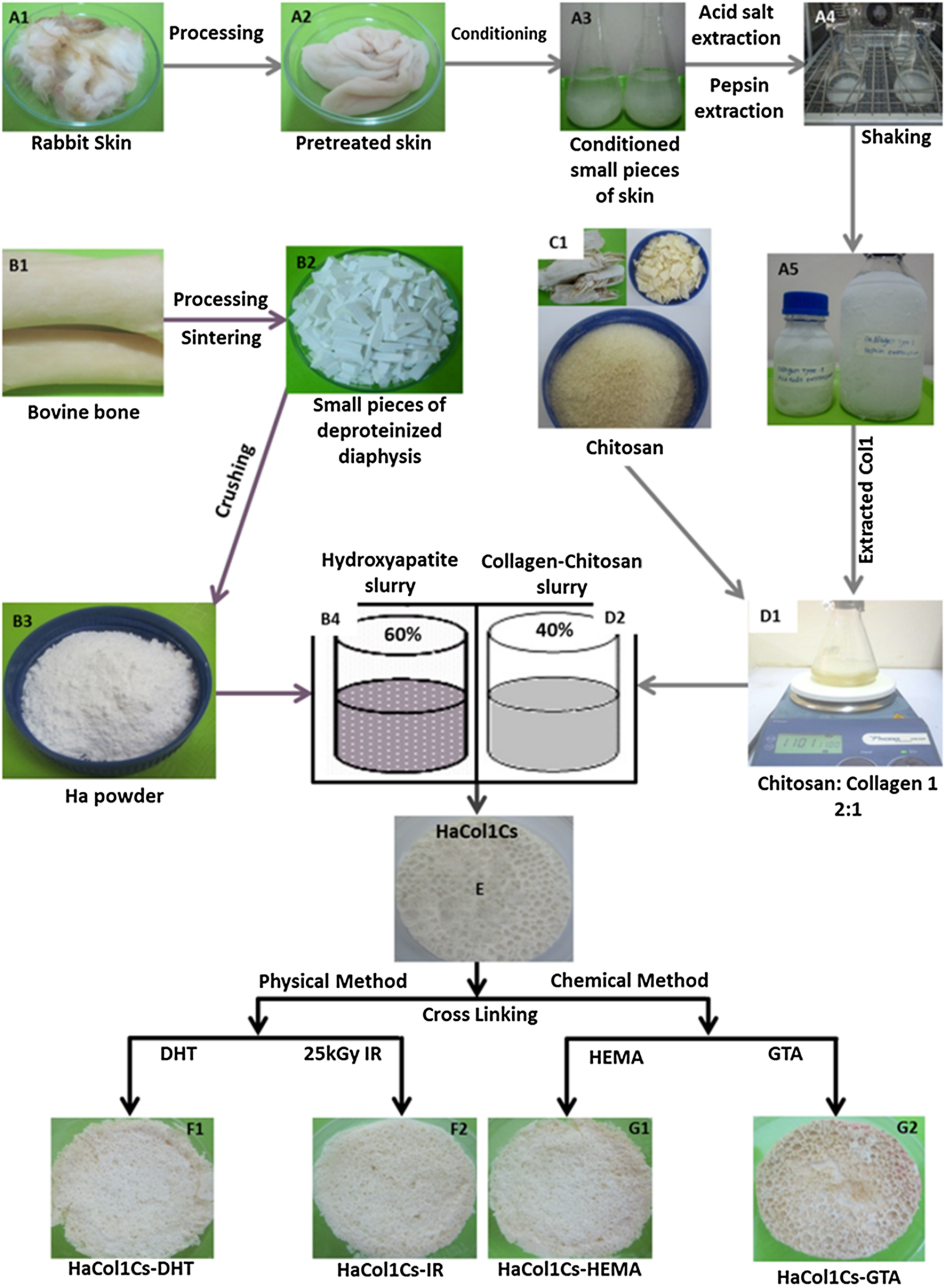
Schematic representation for the fabrication of porous Ha·Col1·Cs scaffold. (A1–A5) Isolation steps of Col1. (B1–B3) Extraction and processing of Ha. (B4) Dispersion of HA powders in the water. (C1) Extracted brine shrimp derived Cs. (D1) Mixing of Cs in the Col1 solution at 2:1 ratio. (B4 and D2) Mixture of Ha slurry, and Cs-Col1 solution at 60:40 ratio and homogenization. (E) Freeze-dried Ha·Col1·Cs scaffold without cross-linkers. Cross-linked fabricated scaffold with physical method (DHT and IR) (F1-F2) and chemical method (HEMA and GTA) (G1-G2)

The manufacturing process of Ha·Col1·Cs scaffold as described in the method section led to a stable co-precipitated Ha·Col1·Cs composite consisting of Ha, Col1 and Cs. To optimize the molecular links between the constituents, the composite material was chemically and physically cross-linked. Four types of scaffolds Ha·Col1·Cs-IR, Ha·Col1·Cs-GTA, Ha·Col1·Cs-DHT, and Ha·Col1·Cs-HEMA were prepared using physical cross-linkers DHT and IR; and chemical method cross-linking with HEMA and GTA solution (Fig. 1). The characterization of in-house-extracted hydroxyapatite and collagen type 1 has been provided in supplementary results (Figures S1, S2).

### Fourier-transform infrared spectroscopy (FTIR) analysis of the scaffolds

**Fig. 2:**
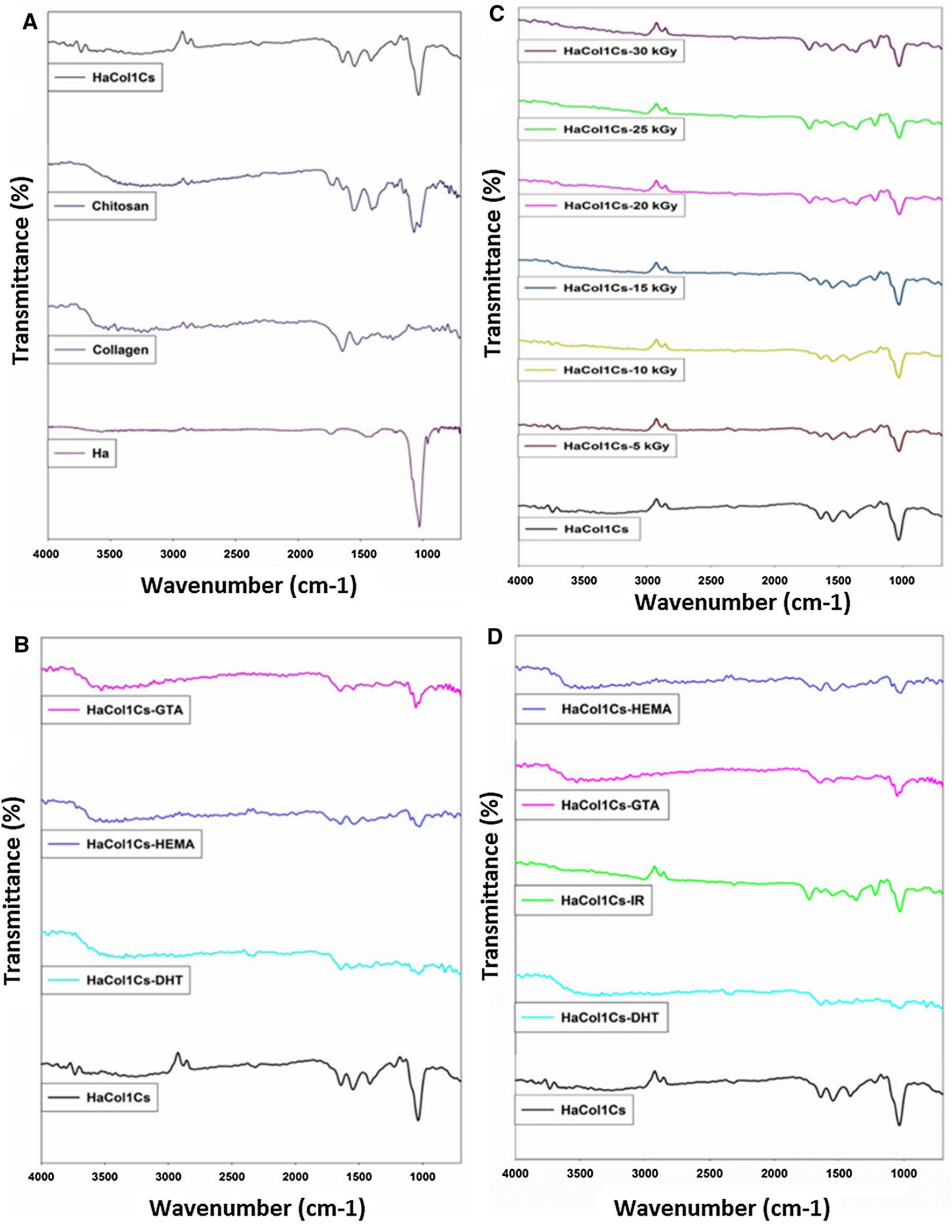
FTIR spectra of Ha·Col1·Cs scaffolds with and without cross linkers. **a** Ha·Col1·Cs (without cross-linkers) spectra with corresponding individual constituents namely Ha, Cs and Col1. **b** FTIR analysis for Ha·Col1·Cs-DHT, Ha·Col1·Cs-HEMA, and Ha·Col1·Cs-GTA where Ha·Col1·Cs (without cross-linkers) served as reference. **c** Effects of various radiation doses on Ha·Col1·Cs scaffold as a cross-linker. **d** Comparative analysis of FTIR spectra between Ha·Col1·Cs (without cross-linkers), Ha·Col1·Cs-DHT, Ha·Col1·Cs-IR (25 kGy), Ha·Col1·Cs-HEMA and Ha·Col1·Cs-GTA

FTIR analysis revealed the usefulness of chemical and physical cross-linker in the scaffold to form better network (Fig. 2). The FTIR spectra of native Ha clearly exhibit peaks at 602, 962, and 1035 cm^−1^, corresponding to PO_4_^−3^ ion. A small and sharp band was detected at 3572 cm^−1^, corresponding to the stretching mode of the –OH group, which results from hydrated calcium phosphate such as Ha. A weak peak was observed at 876 cm^−1^ and strong peak at 1450 cm^−1^ which correspond to the stretching vibration of CO_3_^2−^ ions confirming that Ha crystals containing CO_3_^2−^. The characteristic bands for HPO_4_^2−^ were shown at 1133 cm^−1^, 1064 cm^−1^, 989 cm^−1^, 577 cm^−1^ and 527 cm^−1^. Similarly, the broad bands at about 3200 and 2800 cm^−1^ corresponded to the absorbed hydrate ion and short peaks in the range 3570–3670 cm^−1^ belong to the stretching vibrations of lattice OH– ions of hydroxyapatite (Fig. 2a).

For chitosan, the spectrum around 3430 cm^−1^ attributed to the pooled stretching vibration of OH and N–H groups. The band at 2845 cm^−1^ corresponded to the CH bond stretching. The comprehensive bands at 1637 and 1543 cm^−1^ were assigned to the existence of amide I and amide II groups. The sharp band at 1408 cm^−1^ was attributed to the stretching of carbonyl from the COO^−^ group. The low intense bands at 1382 and 1321 cm^−1^ were attributed to the CH bending vibrations of the ring. The featured peaks of C–O–C glycosidic linkage were shown at the region of 1153–1021 cm^−1^ (Fig. 2a). FTIR of collagen type 1 detected bands of amide A (3299 cm^−1^), amide B (2950–2919 cm^−1^), amide I (1632–1664 cm^−1^), amide II (1500–1585 cm^−1^) and amide III (1200–1300 cm^−1^) (Fig. 2a).

The main absorption bands of Ha·Col1·Cs scaffolds (without cross-links) are amide A (3299 cm^−1^), amide B (2950–2919 cm^−1^) and amide I (1632–1664 cm^−1^) with N–H stretching signature. Amide II (1500–1585 cm^−1^) and amide III (1200–1300 cm^−1^) were also detected in the composite. A free N–H stretching vibration is present between 3400 and 3440 cm^−1^. When the NH group of a peptide is evolved in a hydrogen bond, this position is moved to around 3300 cm^−1^. The characteristic bands for OH– appeared at 3452–3782 cm^−1^. Other functional groups such as amide III, PO_4_^3−^*V*_3_, and asymmetric HPO_4_^2−^ were detected (Fig. 2a).

In this study, carbonate *V*_2_ was identified in sample Ha·Col1·Cs-DHT, Ha·Col1·Cs-IR and Ha·Col1·Cs-GTA at 824–870 cm^−1^, 898 cm^−1^ and 897 cm^−1^ sequentially. CO_3_*V*_3_ was detected in the samples Ha·Col1·Cs (1412, 1543 cm^−1^), Ha·Col1·Cs-DHT (1402, 1560 cm^−1^), Ha·Col1·Cs-IR (1415, 1549 cm^−1^) and Ha·Col1·Cs-GTA (1397, 1545 cm^−1^). Amide I was found for samples Ha·Col1·Cs, Ha·Col1·Cs-DHT, Ha·Col1·Cs-IR and Ha·Col1·Cs-GTA at 1639, 1643 cm^−1^; 1641 and 1645 cm^−1^ correspondingly. The N–H stretching was also shown for sample Ha·Col1·Cs, Ha·Col1·Cs-DHT, Ha·Col1·Cs-IR and Ha·Col1·Cs-GTA at 3213, 3398 cm^-1^; 3266 cm^−1^; 3123, 3572 cm^−1^; and 3185, 3526 cm^−1^, respectively. Besides this, other functional groups such as amide III, PO_4_*V*_3_ and asymmetric HPO_4_^2−^ were found (Fig. 2b).

In case of various irradiated samples (Fig. 2c), FTIR spectra showed a band of amide I stretching at ~ 1635 cm^−1^for Ha·Col1·Cs without cross-linking and for the irradiated scaffolds (5–20 kGy). However, a high radiation dose (25 and 30 kGy) shifted the band to 1643 cm^−1^. The amide II stretch at 1543 cm^−1^ was stable in all of the scaffolds, which indicated that radiation has no impact on amide II bond.

When HEMA was cross-linked to Ha·Col1·Cs, C=O stretching vibration was observed to shift from 1720 to 1608 cm^−1^ due to amide II of collagen. The interaction of the alkane group with the –OH group of HEMA shifted the –OH group. The C–O stretching of HEMA was visible due to its shift from 1153 to 1091 cm^−1^ because of cross-linking with amide III of collagen (Fig. 2d).

### Morphology analysis of the scaffolds from scanning electron microscopy (SEM) image

**Fig. 3:**
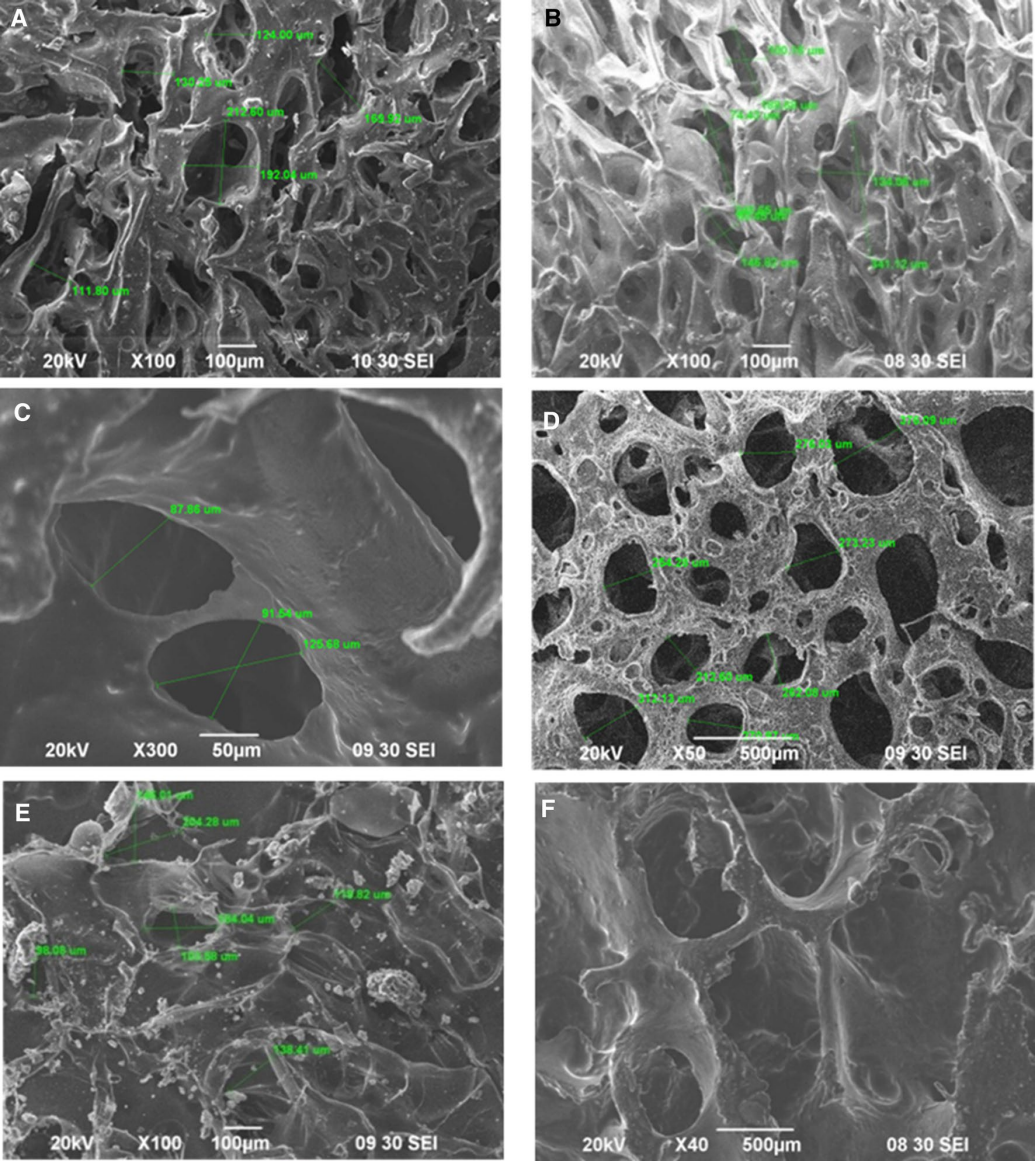
SEM micrographs of different Ha·Col1·Cs scaffolds and human bone graft (HBG) from horizontal cross-sections at the middle region of the scaffold. **a** Ha·Col1·Cs scaffold without cross-linked. **b** Ha·Col1·Cs-IR scaffold cross-linked with 25 kGy gamma irradiation. **c** Ha·Col1·Cs-DHT scaffold cross-linked with DHT method. **d** Ha·Col1·Cs-GTA scaffold cross-linked with GTA solution. **e** Ha·Col1·Cs-HEMA scaffold cross-linked with HEMA. **f** Human bone graft served as positive control

SEM images showed that all scaffolds exhibited irregular porous structures with moderate interconnections among the pores (Fig. 3). The wall of the macro-pores was detected to contain micro-pores. The pore diameter was 111.8–212.6 µm (mean 156.77 µm ± 37) for the Ha·Col1·Cs composite (Fig. 3a). On the other hand, the pore diameter of Ha·Col1·Cs-IR, Ha·Col1·Cs-DHT, Ha·Col1·Cs-GTA and Ha·Col1·Cs-HEMA were 74.43–341.12 µm (mean 164.3 µm ± 86) (Fig. 3b), 87.86–125.68 µm (mean 101.69 µm ± 17) (Fig. 3c), 212.6–376.09 µm (mean 273.43 µm ± 49) (Fig. 3d) and 98–204 µm (mean 142 µm ± 40) (Fig. 3e), respectively.

### Porosity, density, stability, mechanical strength and degradation study

**Fig. 4:**
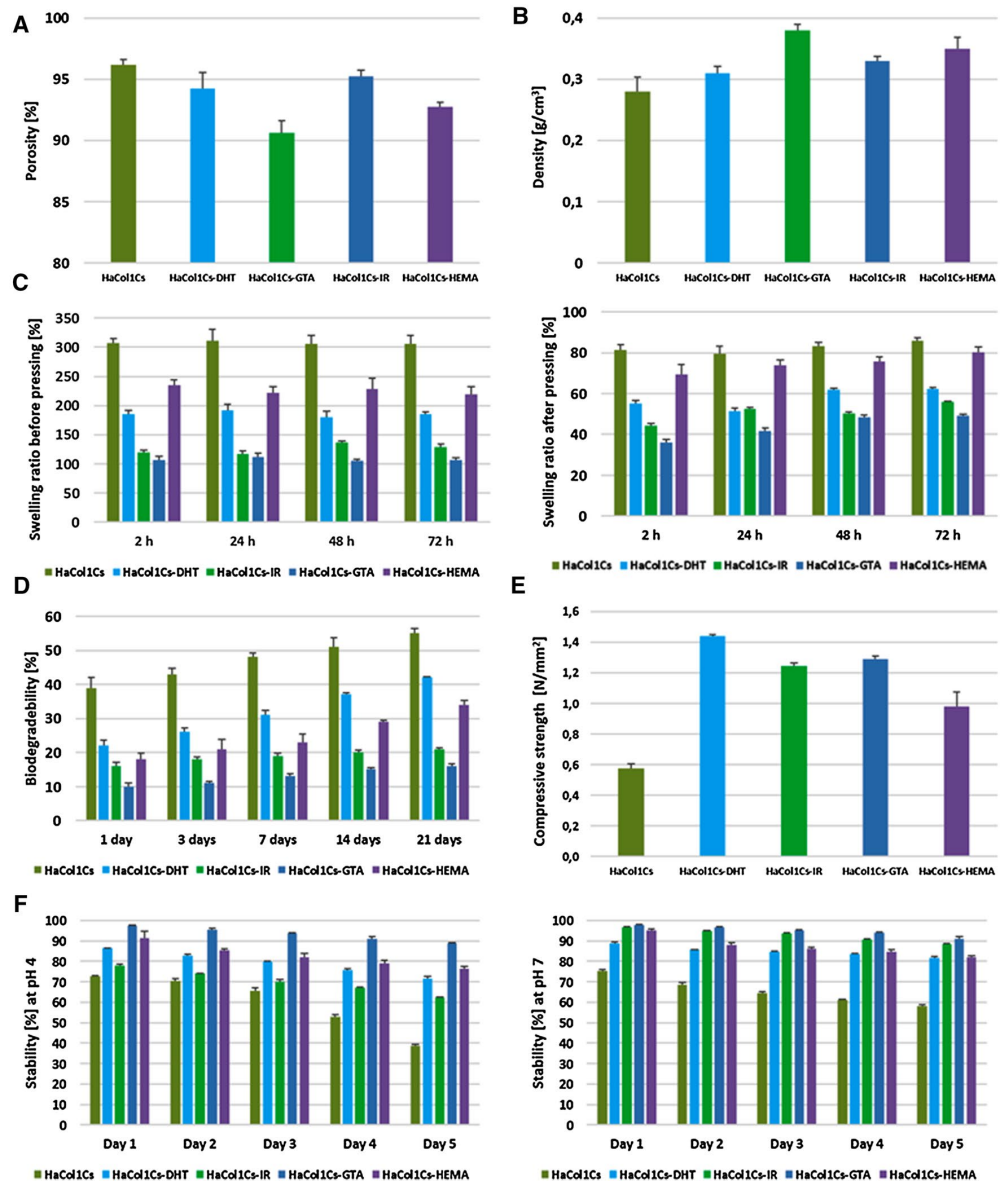
Physicochemical characterization of the fabricated scaffolds. **a** Porosity range of the scaffolds. **b** Density of the fabricated scaffolds. **c** Swelling percentage of Ha·Col1·Cs (non-cross-linked and cross-linked) scaffolds at different soaking time: (left) swelling percentage of scaffold composition on the overall water uptake and (right) swelling percentage of scaffold material itself. **d** Enzymatic degradation studies of Ha·Col1·Cs (non-cross-linked and cross-linked) scaffolds. **e** Mechanical strength. **f** Stabilities of Ha·Col1·Cs scaffolds (non-cross-linked and cross-linked) in aqueous solution: (left) stability test at pH 4.0 and (right) stability test at pH 7.0

The porosity and density of the fabricated scaffolds were measured by liquid displacement test (LDT) using ethanol as a fluid. Ha·Col1·Cs-DHT, Ha·Col1·Cs-IR and Ha·Col1·Cs-GTA scaffolds showed porosities of 94.24, 95.29 and 90.64%, in the order given. The highest porosity (96.21%) was found in Ha·Col1·Cs without any cross-linker (Fig. 4a). Among the samples, the highest density was found for Ha·Col1·Cs-GTA (0.38 g/cm^3^) and the lowest was found for Ha·Col1·Cs without any cross-linker (0.28 g/cm^3^) (Fig. 4b). The highest swelling ratio was observed for the scaffold of without cross-linking (~ 306.24%) and the lowest was detected for Ha·Col1·Cs-GTA (~ 106.24%) after 72 h (Fig. 4c).

The biodegradation rate also varied between the different scaffolds. The lowest degradation rate was found in Ha·Col1·Cs-GTA which was 10% (day 1) and 16% (day 21). On the other hand, scaffold without cross-linker (Ha·Col1·Cs) showed a faster degradation rate of 39% (day 1) and 55% (day 21) (Fig. 4d).

Mechanical strength test revealed that all cross-linked scaffolds had a higher mechanical strength than the initial Ha·Col1·Cs indicating that, mechanical properties of the scaffold were affected by cross-linking. Among the cross-linked scaffold, Ha·Col1·Cs-DHT showed the highest strength (1.4 N/mm^2^) whereas Ha·Col1·Cs-GTA and Ha·Col1·Cs-IR had values around 1.2 N/mm^2^ (Fig. 4e).

Stability test of the fabricated scaffolds illustrated that scaffolds with cross-linker were more stable than Ha.Col.Cs without linker. In general, samples more stable were lower stable in neutral pH (pH 7.0) than on mild acidic conditions (pH 4.0). In both cases, Ha·Col1·Cs-GTA showed the highest stability (Fig. 4f).

### In vitro cytotoxicity and human blood biocompatibility analysis

**Fig. 5:**
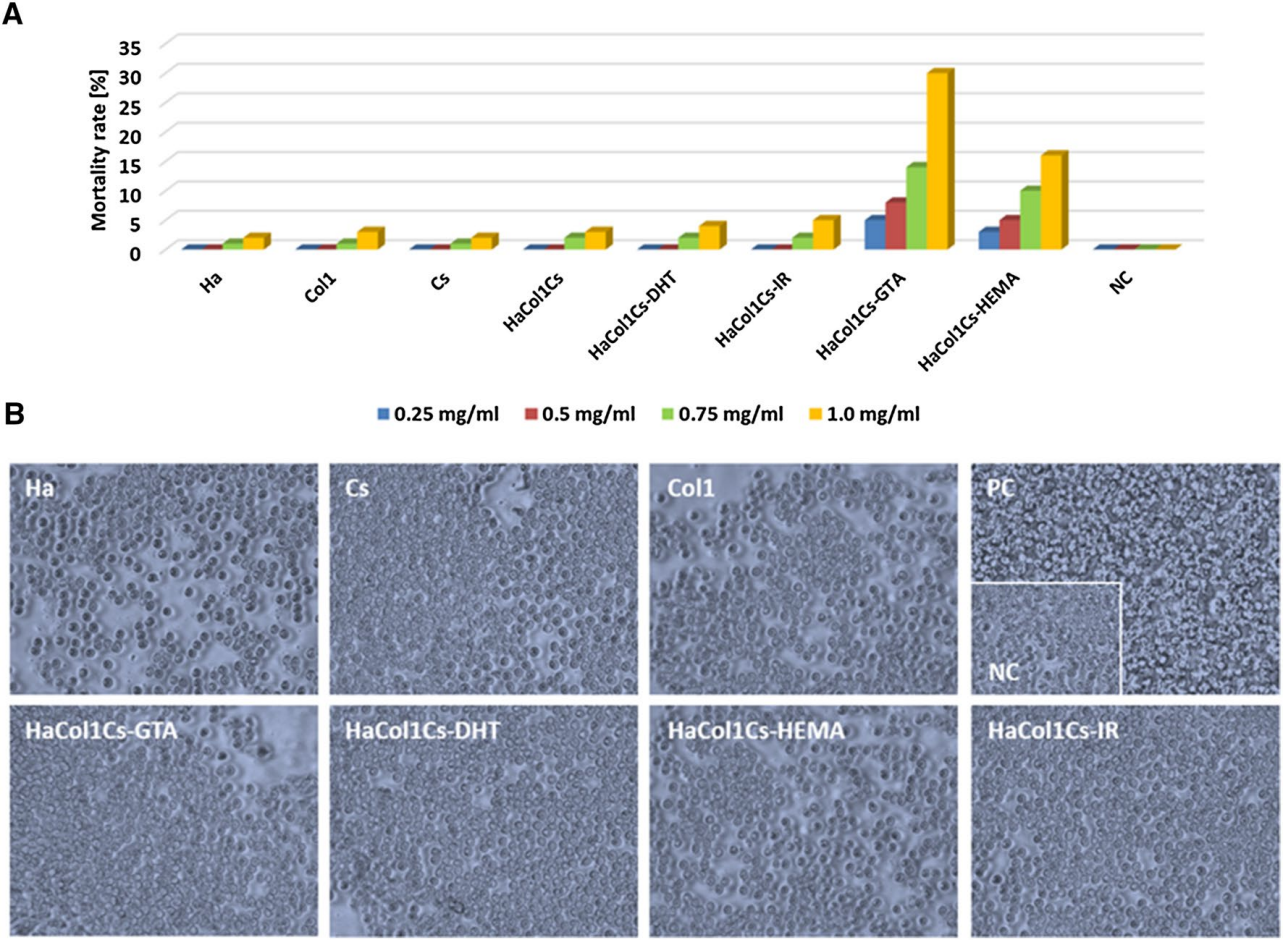
In vitro cytotoxicity and human blood biocompatibility of Ha·Col1·Cs scaffolds and its constituents. **a** Brine shrimp lethality assay. **b** RBC hemolysis biocompatibility assay. PC positive control, and distilled water served as NC negative control

Brine shrimp lethality bioassay revealed that the composite biomaterials constituents (Ha, Cs and Col1) individually and the scaffold Ha·Col1·Cs as well as the physical cross-linked Ha·Col1·Cs-DHT, Ha·Col1·Cs-IR did not have a cytotoxic effect at a concentration of < 1 mg/mL. However, chemical cross-linked scaffold Ha·Col1·Cs-HEMA and Ha·Col1·Cs-GTA were observed to be more lethal than others. In general, the mortality rate was increasing above 1 mg/mL concentration of the scaffolds (Fig. 5a). Biocompatibility was assessed by incubation of individual materials with human red blood cells (RBCs) and showed no adverse effect on RBCs (Fig. 5b).

### AF-MSCs attachment, growth and mineralization observation

**Fig. 6:**
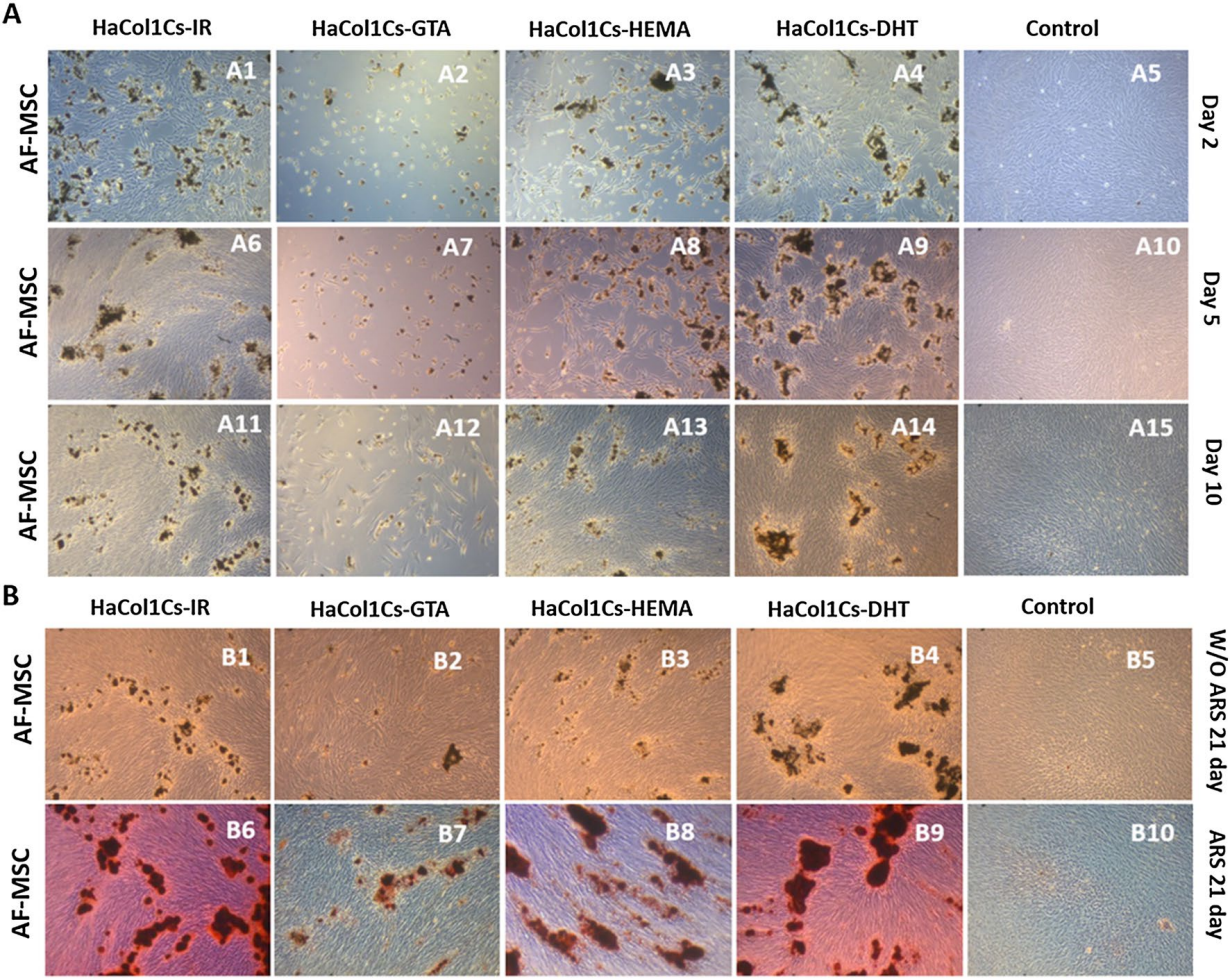
AF-MSC attachment, growth and mineralization analysis in presence of various Ha·Col1·Cs scaffolds. **a** AF-MSC attachment and growth in presence of the formulated scaffolds powder. **b** In vitro mineralization of AF-MSCs in presence of distinct scaffolds. Calcification was evidenced by Alizarin Red (ARS) staining

We demonstrated human AF-MSCs attachment, viability and growth in the presence of the respective scaffolds nano-powder (Fig. 6a). In the presence of Ha·Col1·Cs-IR and Ha·Col1·Cs-DHT, normal cell behavior was observed (Fig. 6A1, A4). In case of Ha·Col1·Cs-HEMA, a lower number of cells attached and the morphology of the cells was changed (Fig. 6A3, A8). However, Ha·Col1·Cs-GTA was observed to be lethal for AF-MSCs leaving almost no living cells after 2 days (Fig. 6A2). These results exhibit the compatibility of the Ha·Col1·Cs-IR and Ha·Col1·Cs-DHT for AF-MSCs. After 5 days a uniform interconnection of MSC network on the scaffolds as well as multiple cell–cell contacts were visualized (Figs. 5A9, 6A6). At day 10, we observed the AF-MSC confluency was more than 90% in the presence of Ha·Col1·Cs-IR (Fig. 6A11), Ha·Col1·Cs-DHT (Fig. 6A14) and Ha·Col1·Cs-HEMA (Fig. 6A13) indicating no negative effect on proliferation. We also noticed that cells adapted to Ha·Col1·Cs-GTA with time, allowing growth of the surviving cells (Fig. 6A12).

Regarding in vitro mineralization in the presence of MSCs, we observed that clusters of MSCs aggregated together around the scaffolds particles and formed boney-like structure (Fig. 6B1–B5). Calcium phosphate deposition by the AF-MSCs was also evidenced by Alizarin Red staining (ARS) in the presence of scaffolds at day 21(Fig. 6B6–B10). An enhanced mineral deposition was found for the composite of Ha·Col1·Cs-IR and Ha·Col1·Cs-DHT compared to Ha·Col1·Cs-HEMA (Fig. 6B6, B9).

### In vivo grafting of Ha·Col1·Cs-IR and Ha·Col1·Cs-DHT into a rabbit mandible defect model

**Fig. 7:**
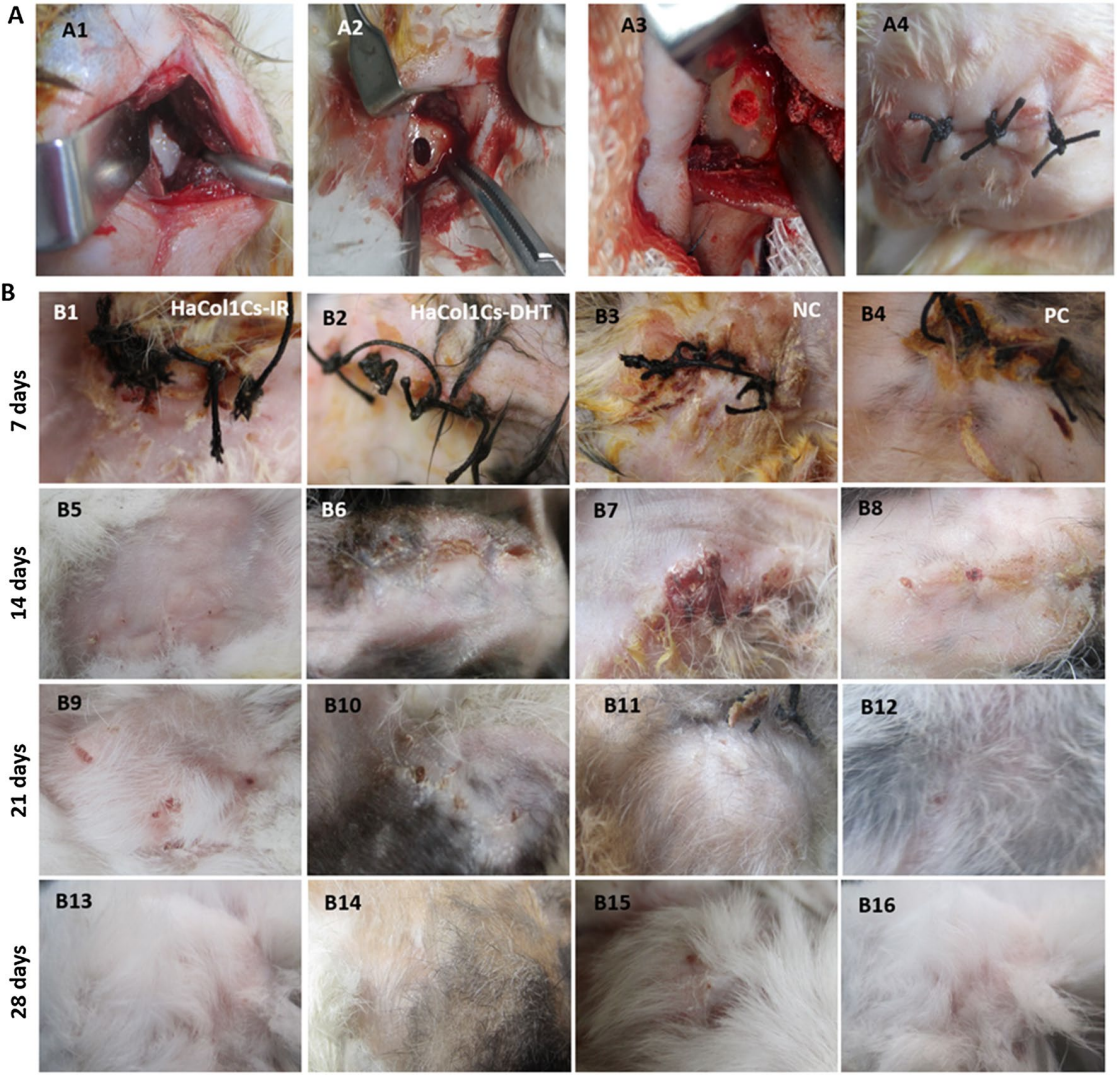
In vivo grafting of scaffold in the surgically created rabbit maxillofacial mandible defect (non-load bearing) model. **a** Surgical and implantation procedures. (A1) Surgical incision showed the site of mandible to be drilled. (A2) Drilled defect chamber in the mandible of rabbits. (A3) Defect filled with scaffold. (A4) Sutured incision. **b** Post-grafting recovery observation from day 7 to day 28

Based on the physicochemical and in vitro biological tests, Ha·Col1·Cs-IR and Ha·Col1·Cs-DHT were qualified as good candidates to be transplanted in a rabbit maxillofacial mandible defect model (Fig. 7a). By 2 h post-operation, the rabbits appeared to be normal with regards to their eating habits and movements. Furthermore, we did not observe any adverse reactions or post-operative complications such as abnormal bleeding or infection at surgical sites. Further, we did not notice any signs of inflammation such as swelling, and the grafted materials were confirmed to be intact within the defects. However, after 4 weeks, the surgical area of each rabbit was healed with minor scar marks and covered with new hair (Fig. 7B13–B16).

### Post-operative histological and radiological analysis

**Fig. 8:**
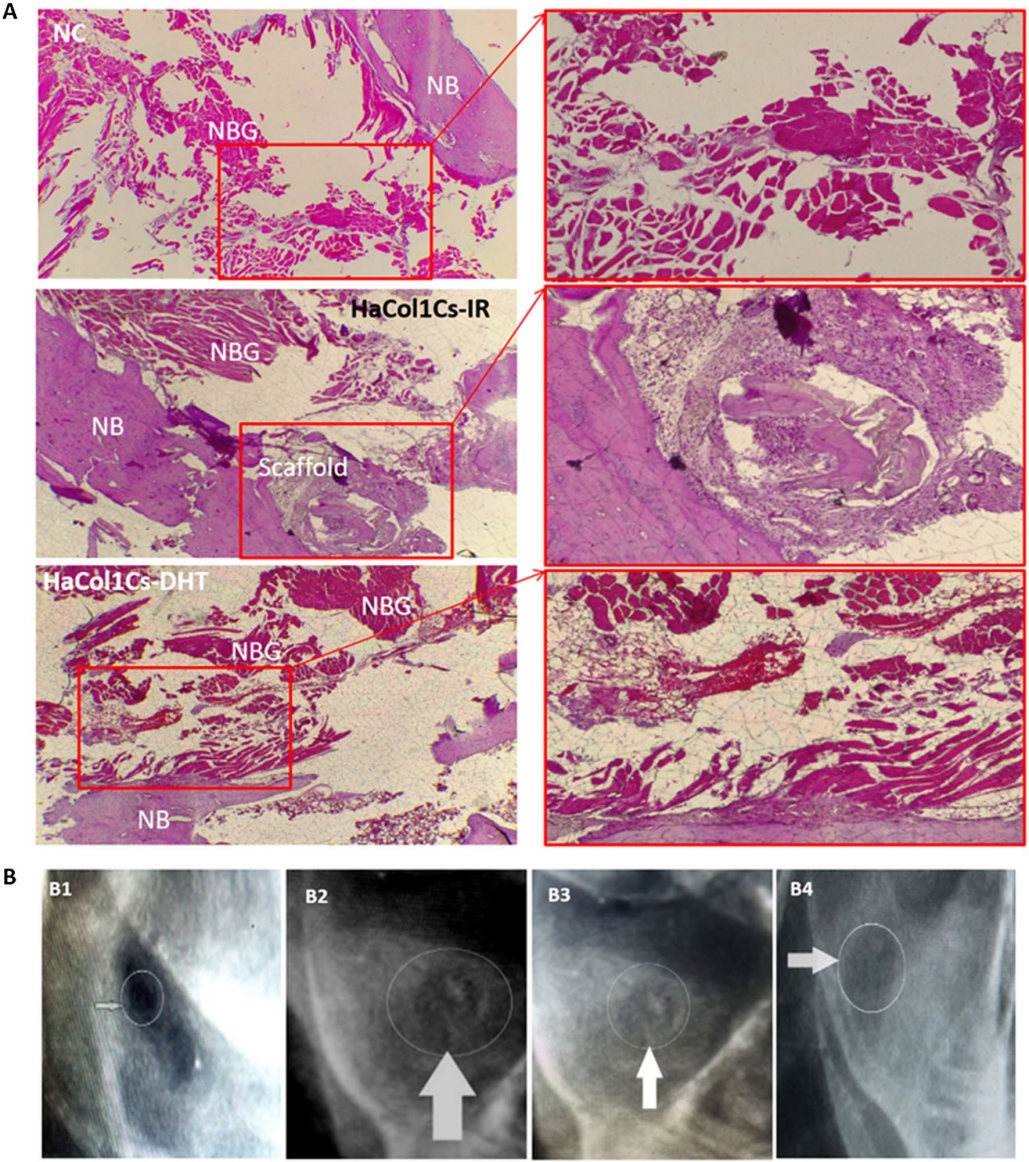
Post-operative histological and radiological analysis. **a** Histological analyses of the treated defects after 4 months. Defected mandible without any implant/graft (first lane), Ha·Col1·Cs-IR (second lane), and Ha·Col1·Cs-DHT (third lane) whereas native bone (NB) and newly bone growth (NBG) are distinguished. **b** Representative radiological images during the recovery period in the Ha·Col1·Cs-IR group from (B1) day 1 to (B4) 4 months

Hematoxylin and eosin (H&E) staining was done to access the comparative histology of the experimented mandible bones which were implanted with Ha·Col1·Cs–IR, Ha·Col1·Cs-DHT, human bone graft (gold standard positive control), and defects without transplantations served as a negative control. No significant differences were observed between Ha·Col1·Cs-DHT and Ha·Col1·Cs-IR concerning the formation of new blood vessels, and new bone structures (Fig. 8a). However, the human bone graft was superior whereas empty controls were inferior when compared to Ha·Col1·Cs-DHT and Ha·Col1·Cs-IR regarding bone regeneration 4 weeks after transplantation. Representative radiological images of the Ha·Col1·Cs-IR transplanted group revealed the gradual regeneration and filling of the defected rabbit mandible (Fig. 8B1–B4).

## Discussion

All biomaterials used in this study were obtained from bio-waste which would have been discarded. As such we have extracted Ha from bovine cortical bone, Col1 from slaughtered rabbit skin and Cs from prawn shell (Fig. 1A1, B1, C1). Thus, we have used materials and procedures which are a biologically safe and economically desirable (Rincón-López et al. 2018; Pachence 1996; El-Jawhari et al. 2016; Khan et al. 2005, 2012). Recently, these constituents for the fabrication of scaffold gained attention encompassing tunable chemical (molding ability into various geometries and formation of porous structures) and biological (suitable for cell growth and osteo-conduction) properties (Wang et al. 2017; Dan et al. 2016; Qasim et al. 2017; Zhang et al. 2013; Demirtaş et al. 2017; Chamieh et al. 2016; Balagangadharan et al. 2017).

In line with our FTIR analysis (Fig. 2a), the major band of the amide I in cross-linked Ha-Col1 sample was focused at 1653 cm^−1^ and minor bands at 1636 and 1663 cm^−1^ (Boskey et al. 1999; Epaschalis et al. 2001). In general, no significant changes were observed in the mineral phase of the scaffold due to radiation; however, new bonds (C–N triple bond and C–C triple bond) were formed in the polymer phase (Fig. 2c). Due to the irradiation, bonds could be formed between the polypeptide chains without utilizing the acidic and basic side chains which control the pore structure. Since, cellular attachment and infiltration are significantly affected by the scaffolds’ mean pore size (Murphy et al. 2010); we have measured this parameter by SEM. The mean pore diameter for all scaffolds was found to range from 98 to 204 µm (Fig. 3), which is similar to pore sizes reported for other microparticle-based scaffolds (100–800 µm) (Reves et al. 2009) and above the required minimum size (50 µm) as needed for osteogenesis (Cheung et al. 2015).

In this study, Ha·Col1·Cs scaffolds were fabricated by thermally induced phase separation technique (Fig. 1e) with good porosity (90.64–96.21%) and water absorption capacity (Fig. 4a, c). The measured porosity reached the recommended porosity of ≥ 90% for bone substitute materials to accommodate osteoblasts or osteoprogenitor cells (Sabir et al. 2002). Therefore, this scaffold was qualified to be good penetrable by cell suspensions, required nutrient, metabolites, and soluble signals. The density of the fabricated scaffold ranged from 0.28 to 0.38 g/cm^3^ (Fig. 4b), whereas the apparent density of trabecular bone was reported to range from 0.14 to 1.10 g/cm^3^ (Evans et al. 1992).

The swelling ability depends on the microstructure and hydrophilic nature of the scaffold (Yan et al. 2010). Since Col1 and Cs are both hydrophilic materials, the fabricated scaffolds were shown to have a relevant swelling ability which is in favor of maintaining the porous structure (Chen et al. 2016). The scaffolds without cross-linker showed relatively lower stability than the cross-linked Ha·Col1·Cs scaffolds (Fig. 4f). It was also revealed that the stability of the Ha·Col1·Cs scaffolds was reduced in acidic condition which has been shown before (Khan et al. 2012). We could show that cross-linking had a profound effect on the mechanical stability of the scaffold. Ha·Col1·Cs-DHT showed the best strength amongst the cross-linked scaffolds (Fig. 4e). Different studies also presented similar compressive strength in the hydroxyapatite-collagen scaffold and showed that the compressive strength of the collagen scaffold was increased by de-hydrothermal treatment (Kozlowska and Sionkowska 2015; Nitzsche et al. 2010).

One feature of a good bone scaffold is that the scaffold and its degradation products should not provoke an inflammatory response (Velasco et al. 2015; Alaribe et al. 2016). The degradation rate of porous scaffold influences cell vitality, cell growth, and even host response (She et al. 2008; Tan et al. 2007). In our study, the biodegradation was best for Ha·Col1·Cs without cross-linking and varied between the different cross-linker methods (Fig. 2e) whereas Ha·Col1·Cs-DHT was better degradable than the others. Aqueous mixtures of collagen and chitosan form electrostatic interactions between positively charged amino groups and negatively charged carboxyl groups leading to a complex structure (Taravel and Domard 1996; Khan et al. 2012). Cross-linking with DHT is supposed to cause the formation of new amide bonds in protein-based materials (Geiger et al. 2003). For this reason, it seems evident that DHT makes collagen more resistant to enzymatic degradation (Wahl and Czernuszka 2006).

The Ha·Col1·Cs-IR exhibited a lower biodegradability than Ha·Col1·Cs-DHT which may be due to the cross-linking method in which high-energy ionizing radiation or photoinitiator molecules are used. This method eventually influences the mechanical properties and degradation behavior of irradiated collagen-based scaffolds (Davidenko et al. 2015; Hovakimyan et al. 2012; Lew et al. 2007).

Our extracted Ha, Col1 and Cs were RBC biocompatible and non-toxic to brine shrimp larvae, which qualify these as biomaterial precursors for scaffold preparation (Khan et al. 2012; Levengood and Zhang 2014). Furthermore, we observed Ha·Col1·Cs-IR and Ha·Col1·Cs-DHT were compatible for AF-MSCs attachment and growth (Fig. 6A1, A4) which may be due to Col1 which is widely used as a coating material and constituent of scaffolds supporting cell attachment (Cooke et al. 2008; Wang et al. 2017; Zhang et al. 2013).

Ha·Col1·Cs-GTA inhibited cell attachment and growth of AF-MSCs (Fig. 6A2, A12). Previously it has been reported that adipose-derived stem cells attached and grew slowly in presence of a GTA–sponge (Yang et al. 2018). Although GTA is a widely used chemical cross-linker, it was reported that the functional aldehyde groups of GTA are toxic for cells (Oryan et al. 2014) and may cause significant biohazard problems, which has limited its application in commercial products (Yoo et al. 2011).

Alizarin red staining provided a proof that the AF-MSCs formed calcium-based mineral deposits around the scaffolds at day 21(Fig. 6b). Mineralization was reported to be induced by the osteoconductive nano-Ha powder present in the composites (Zhang et al. 2014; O’Brien 2011). In line with our work, chitosan–gelatin/nanohydroxyapatite scaffolds have been shown to support MC3T3-E1 cell attachment, proliferation, and mineralization (Dan et al. 2016).

At cellular level, the composite scaffold acts as an impermanent matrix for cell proliferation until new bone tissue is completely regenerated (Wattanutchariya and Changkowchai 2014). In vivo compatibility and utility of Ha·Col1·Cs-IR and Ha·Col1·Cs-DHT were evaluated (Fig. 7). We observed that both compatible contributed to mandible bone restoration. This regeneration capacity was superior to non-treatment of the defect but was inferior to the gold standard bone graft (Fig. 8). In line with our study, it was previously demonstrated that the nHAC/PLGA scaffolds implanted rabbit critical-size mandible defect possessed tissue compatibility and higher bone restoration capacity compared to empty controls (Wang et al. 2017).

Here, we did not add any growth factors, or cells to the scaffolds. By addition of bone-marrow MSCs to nanohydroxyapatite/collagen/poly l-lactide scaffolds total bone formation in a rabbit critical-size mandibular bone defect model was significantly higher than without the addition of stem cells (Wang et al. 2016). Therefore, for optimal bone healing, a combination of stem cells and biomaterials was reported to be needed to treat periodontal bone defects (Wang et al. 2017), suggesting the necessities of additional studies including mesenchymal cells. Additionally, more studies should aim to reveal the regulatory mechanisms involved in the complex process of biomineralization in vivo.

## Conclusion

We have successfully fabricated Ha·Col1·Cs scaffolds from low cost and locally available polymeric bioactive materials using thermally induced phase separation technique with the cross-linkers such as GTA, DTH, IR and HEMA. All four formulated scaffolds showed substantial physicochemical and morphological features. Preliminary in vitro tests on AF-MSCs identified Ha·Col1·Cs-DHT and Ha·Col1·Cs-IR scaffolds as the most competent materials. In vivo, Ha·Col1·Cs-IR and Ha·Col1·Cs-DHT scaffolds significantly supported new bone formation in a maxillofacial mandible defect model making these scaffolds promising for the use in treatment of bone defects. However, 3D porous scaffold design, in vivo transplantation and clinical applications are still requiring significant improvement to harness optimum applicability. In-depth understanding of the basic fabrication processes involved and the post-transplantation mechanisms may help to achieve clinical relevance.

## Electronic supplementary material

Below is the link to the electronic supplementary material.

Supplementary material 1 (TIFF 1639 kb)

Supplementary material 2 (TIFF 436 kb)

Supplementary material 3 (DOCX 19 kb)

Supplementary material 4 (DOCX 2093 kb)

## Abbreviations

Ha: Hydroxyapatite
Col1: Collagen type 1
Cs: Chitosan
SEM: Scanning electron microscopy
HaCol1Cs: Hydroxyapatite-collagen type 1-chitosan
XRD: X-ray diffraction
XRF: X-ray fluorescence spectroscopy
HPLC: High-performance liquid chromatography
SDS-PAGE: Sodium dodecyl sulfate polyacrylamide gel electrophoresis
FTIR: Fourier-transform infrared spectroscopy
GTA: Glutaraldehyde
DHT: De-hydrothermal treatment
IR: Irradiation
TIPS: Thermally induced phase separation
kGy: Kilo gray
DT: Denaturation temperature
HEMA: 2-Hydroxyethyl Methacrylate
Co60γ: Cobalt sixty gamma
LDT: Liquid displacement technique
TGA: Thermal gravimetric analyzer
PBS: Phosphate buffer saline
TFA: Trifluoroacetic acid
n-β-TCP: Nano-β-tricalcium phosphate/collagen
DW: Distilled water
RT: Room temperature
PSC: Pepsin soluble collagen
ASC: Acid soluble collagen
PLGA: Poly lactic-co-glycolic acid
AF-MSCs: Amniotic fluid-derived mesenchymal stem cells
ARS: Alizarin red staining
