# Supplementary figures and images for: Fabrication of biocompatible porous scaffolds based on hydroxyapatite/collagen/chitosan composite for restoration of defected maxillofacial mandible bone
Source: Prog Biomater. 2019 May 29;8:137–54. doi: 10.1007/s40204-019-0113-x (PMC6825626; doi:10.1007/s40204-019-0113-x)

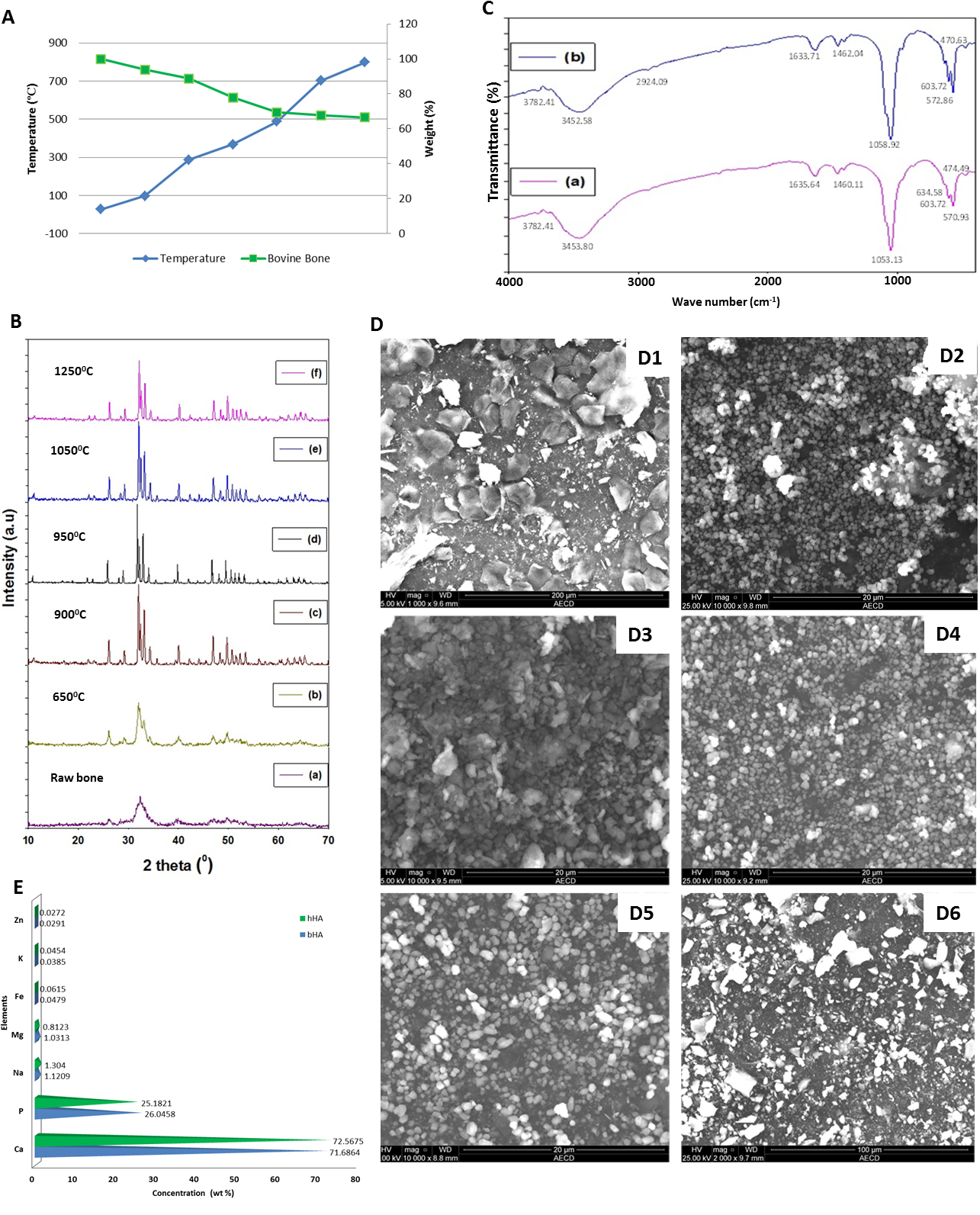

Supplement: Supplementary file 1 — Supplementary material 1 (TIFF 1639 kb) [file 40204_2019_113_MOESM1_ESM.tif]

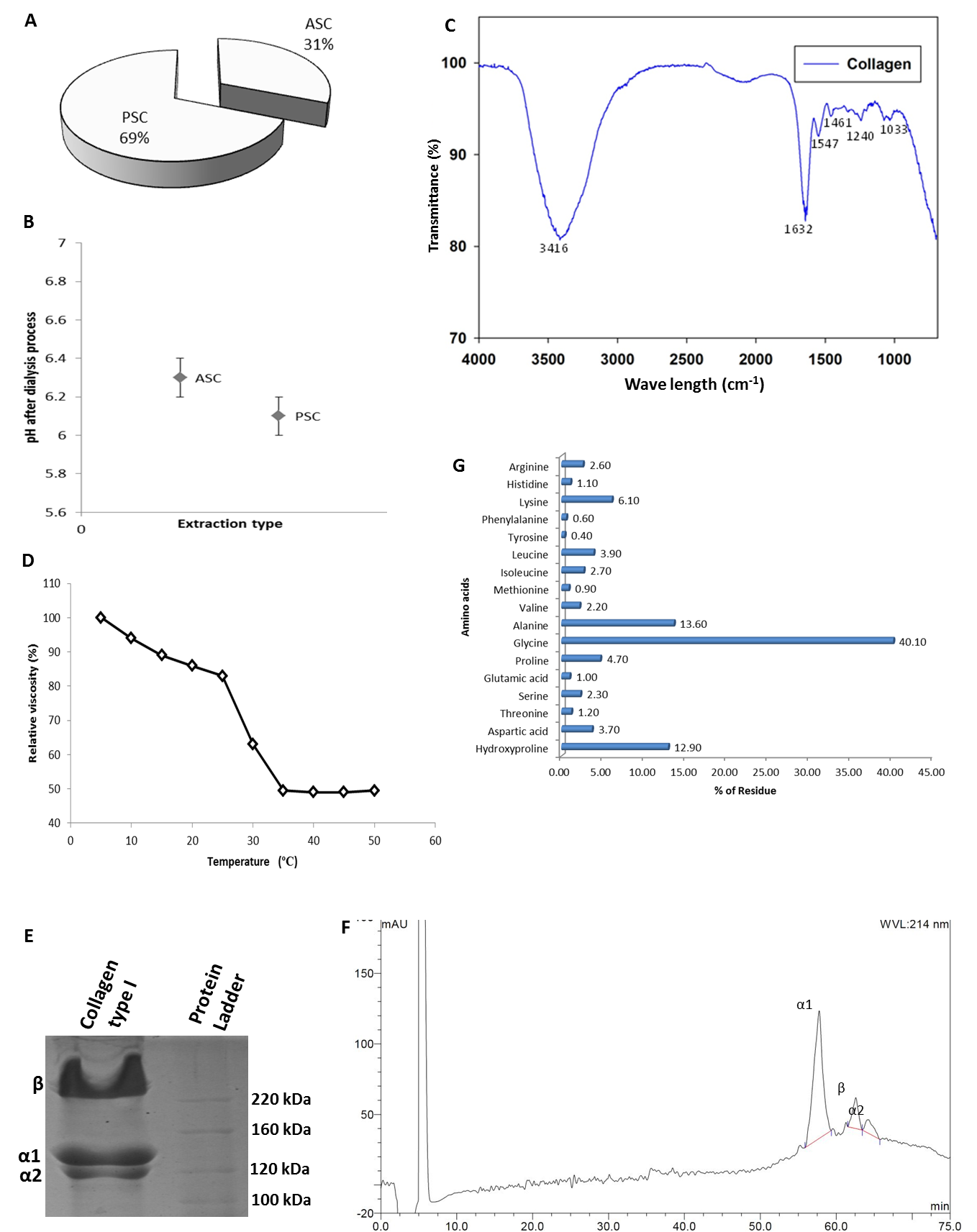

Supplement: Supplementary file 2 — Supplementary material 2 (TIFF 436 kb) [file 40204_2019_113_MOESM2_ESM.tif]
