# Supplementary material for: Fabrication of biocompatible porous scaffolds based on hydroxyapatite/collagen/chitosan composite for restoration of defected maxillofacial mandible bone
Source: Prog Biomater. 2019 May 29;8:137–54. doi: 10.1007/s40204-019-0113-x (PMC6825626; doi:10.1007/s40204-019-0113-x)
Supplement: Supplementary file 3 — Supplementary material 3 (DOCX 19 kb) [file 40204_2019_113_MOESM3_ESM.docx]

**Material and Methods_ Supplementary S1**

**Composite materials extraction**

The diaphysis of the bovine bones was collected for Ha extraction. Briefly, the diaphysis was boiled in deionized water for about 8hrs to remove the bone marrow and tendons and cut into small pieces. After that, the pieces of bone were de-proteinized by boiling. The boiled bone samples were dried overnight at 100°C. Then, the dried samples were annealed in an electric furnace (Borel, 1600, Switzerland), under ambient condition at four different temperatures ranging from 650°C to 1250°C, using a heating rate of 5^0^C/min with 3hrs holding time consequentially as multi stage sintering. The sintered products were crashed with mortar and pestle, and sieved with mesh no. 200. Finally, samples were irradiated at 25kGy for sterilization using a Co60γ source.

Rabbit skin collagen was isolated according to the method described before with some modifications [1]. Pretreated rabbit skins were cut into small pieces (approx. 1cm^2^ each) and suspended in 0.5M acetic acid solution at a solid-to-solvent ratio of 1:10 (w/v). The sample was placed in a shaking incubator (Phoenix Instruments Inc., USA) at 200rpm for 24hrs at RT. After this time, the sample was filtered and the liquid soluble portion was called ASC (Acetic acid soluble collagen). The non-dissolved residue was used for PSC (Pepsin soluble collagen) extraction as follows: the residues were re-suspended in a new solution of 0.5M acetic acid at a concentration of 1:10 (w/v) and pepsin at a concentration of 2mg/ml. This solution was placed in a shaker machine at the same speed as described above for 48hrs. All samples were filtered and stored at 4°C at the end of every single extraction. ASC and PSC were precipitated with a solution of 2.6M NaCl. The precipitated material was centrifuged at 10,000rpm (Model 6800, Kubota, Japan) for 30min. Then, these samples were re-suspended in five volumes of 0.5M acetic acid and dialysed for 48hrs in DW and dialysis membrane tubing with 6–8kDa Mw cut-off. Finally, the resulted dialyzed protein sample was freeze-dried at -55°C.

Chitosan was extracted from shrimp shells according to the method established by Khan et al [2]. Briefly, waste prawn shells were washed with hot DW and dried in an oven at 105^0^C for 72hrs. Dried shells were ground using a blender and deproteinized with 3%NaOH and demineralized with 3%HCl. Then the deproteinized and demineralized chitin was neutralized and dried in oven at 105°C for 24hrs. Chitosan was obtained by deacetylation of chitin with 50%NaOH where the ratio of chitin: NaOH ¼ was 1:20 (w/w) at 100°C for 3hrs. After this process, solids separated from the alkali layer were extensively washed with distilled water to remove traces of alkali. The resultant solid was dried in vacuum oven at 50°C for 24hrs. Here in this work we did not characterize the isolated Cs because it has been already well characterized by one of the group at our organization [3, 4].

**Thermo-gravimetric analysis (TGA) of Ha**

Mass loss pattern of Ha nanopowder during heating were studied by using a TGA analyzer (TGA analyzer, Model Q600, USA). The thermo-gravimetric analysis of the samples during heating were recorded from 30°C to 800°C at a heating rate of 10°C/min in a continuous flow of nitrogen.

**Determination of denaturation temperature (DT) of Col1**

The denaturation temperature (DT) was measured from the viscosity changes according to a described method with some modification [1]. Five hundred ml of 0.03% collagen solution in 0.5M acetic acid were subjected to viscosity measurements using a viscometer (DV2T Touch Screen Viscometer, Brookfield Eng Labs Inc., USA.) with spindle no. 3 and speed of 300 rpm. The thermal determination curve was obtained by measuring solution viscosity at several temperatures ranging from 5°C to 50°C with a heating rate of 5°C/min. At the designated temperature, the solution was maintained for 30min prior to viscosity determination. Measurement was carried out in triplicate. The relative viscosity was calculated in comparison to that obtained at 5°C.

**SDS-PAGE of the extracted Col1**

To determine molecular weight (Mw), sodium dodecyl sulfate polyacrylamide gel electrophoresis (SDS–PAGE) was performed according to the Laemmli and Quittner method with minor modifications [5]. One ml of dialyzed collagen was dissolved in bolt LDS sample buffer (Life Technologies, USA) and boiled for 5min. Then, 10μl of the denatured sample and 8μl of marker with a molecular weight from 10kDa to 220kDa (BenchMark Protein Ladder, Life Technologies, USA) were loaded into wells of the polyacrylamide gel. This gel contained of a 4% stacking gel on top of 10% resolving gel. After migration of proteins, the gel was stained with 0.25% Coomassie brilliant blue for 30 min in 45% methanol and 10% acetic acid (v/v), and then it was de-stained with 40% methanol and 7% acetic acid (v/v) and DW.

**Amino acid composition detection of Col1 using HPLC**

Amino acid analysis was performed using HPLC by the method of Huesgen with required modifications [6]. Col1 was dissolved in 6N HCl and subjected to hydrolysis in boiling water bath at 110°C for a period of 24hrs. The tubes were cyclo-mixed for every 1hr for proper hydrolysis to take place. After 24hrs of hydrolysis, the tubes were centrifuged at 3500rpm for 15min. The supernatant was filtered and neutralized with 1N NaOH. Then the filtered solution was diluted to 1:100 of volume (1ml diluted to 100ml) with milli-Q water and loaded onto HPLC. HPLC analysis was carried out using Dionex Ultimate 3000 LC System (Thermo Scientific, USA). Each sample (1μl) was loaded on a Zorbax 80 A C18 column at 40°C with detection at 338 and 262nm. Mobile phase A was 7.35mM/l sodium acetate/triethylamine (500:0.12, v/v), adjusted to pH 7.2 with acetic acid, while mobile phase B (pH 7.2) was 7.35mM/l sodium acetate/methanol/ acetonitrile (1:2:2, v/v/v). The amino acid composition was expressed as percentage of protein. Data acquisition, data handling, and instrument control were performed by Chromeleon Auto 6.8 Professional software. The columns used were an AcclaimTM 120, C18 (4.6 x 250 mm) column (Thermo Scientific, USA). Gradient elution was carried out with a mixture of two solvents. Solvent A consisted of 0.1% TFA in water, and solvent B was 0.08% TFA in acetonitrile. The flow rate was 1.0ml/min. The stationary phase temperature was kept at 25°C, and detection was carried out at 214nm. The injection volume was 50μl. Proteins were dissolved in water at a concentration of 0.05mg/ml. The fractions, collected in polyethylene tubes, were dried in a centrifuge evaporator.

**Reference:**

[1] Kittiphattanabawon P, Benjakul S, Visessanguan W, Nagai T, Tanaka M. Characterisation of acid-soluble collagen from skin and bone of bigeye snapper (Priacanthus tayenus). Food Chem 2005;89(3):363–372. doi:10.1016/j.foodchem.2004.02.042

[2] Khan MA, Ferdous S, Mustafa AI. Improvement of Physico-mechanical Properties of Chitosan Films by Photo-curing with Acrylic Monomers. J Polym Environ 2005;13:193.

[3] Khan MA, Rahman MA, Khan RA, Rahman N, Islam JMM, Alam R, Mondal MIH. Preparation and Characterization of the Mechanical Properties of the Photocured Chitosan/Starch Blend Film. Polym Plast Technol Eng 2010;49(7)

[4] Rashid TU, Rahman MM, Kabir S, Shamsuddin SM, Khan MA. A new approach for the preparation of chitosan from γ-irradiation of prawn shell: effects of radiation on the characteristics of chitosan. Polym Int 2012;61(8):1302–1308.

[5] Laemmli UK, Quittner SF. Maturation of the head of bacteriophage T4: IV. The proteins of the core of the tubular polyheads and in vitro cleavage of the head proteins. Virology 1974;62(2):483–499. doi:10.1016/0042-6822(74)90409-7

[6] Huesgen G. Sensitive and reliable amino acid analysis in protein hydrolysates using the HP1100 series HPLC. Hewlett Packard Technical Note 1998;12:5966–3110.
