# Supplementary material for: Fabrication of biocompatible porous scaffolds based on hydroxyapatite/collagen/chitosan composite for restoration of defected maxillofacial mandible bone
Source: Prog Biomater. 2019 May 29;8:137–54. doi: 10.1007/s40204-019-0113-x (PMC6825626; doi:10.1007/s40204-019-0113-x)
Supplement: Supplementary file 4 — Supplementary material 4 (DOCX 2093 kb) [file 40204_2019_113_MOESM4_ESM.docx]

**Supplementary file S2_Results**

**Analysis of Ha nano-powder extracted from bovine bone**

During multi stage annealing at distinct temperatures, between 600°C and 800°C, no significant weight loss was observed from TGA (figure S1A). XRD diffraction patterns showed the phase and purity of derived Ha crystals. Ha peaks became sharper and more distinct at 950°C exhibiting the highest diffraction intensities corresponding to the Ha phase compared to all other sintered samples, thus indicating that a highly crystalline Ha structure was obtained at 950°C (figure S1B).

FTIR spectrum values of extracted Ha at 950°C were compared with commercially synthetic Ha (Sigma CAS No.12167-74-7) and detected a large number of bands similarities in the spectra (470, 572, 603, 1058, 1462, 1633, 2854, 2924, 3452, 3782cm^-1^). FTIR spectra of Ha confirmed the presence of a carbonated group and the similarities to commercial Ha (figure S1C). Annealed at 950^0^C, the particle sizes were determined 195.5-339.8 nm by SEM whereas the average particle size was of 255.28 ±63nm (figure S1D4). However, SEM micrograph of the Ha powder showed that the particles had irregular shapes including small spheres and agglomerated together in some parts (figure S1D). XRF analysis revealed that calcium and phosphorus were the main components and magnesium, sodium were the minor elements in which some trace elements (iron, potassium and zinc) were also present (figure S1E). For sterilization of Ha, the samples were exposed in 25kGy and no significant changes were found between initial and irradiated samples.


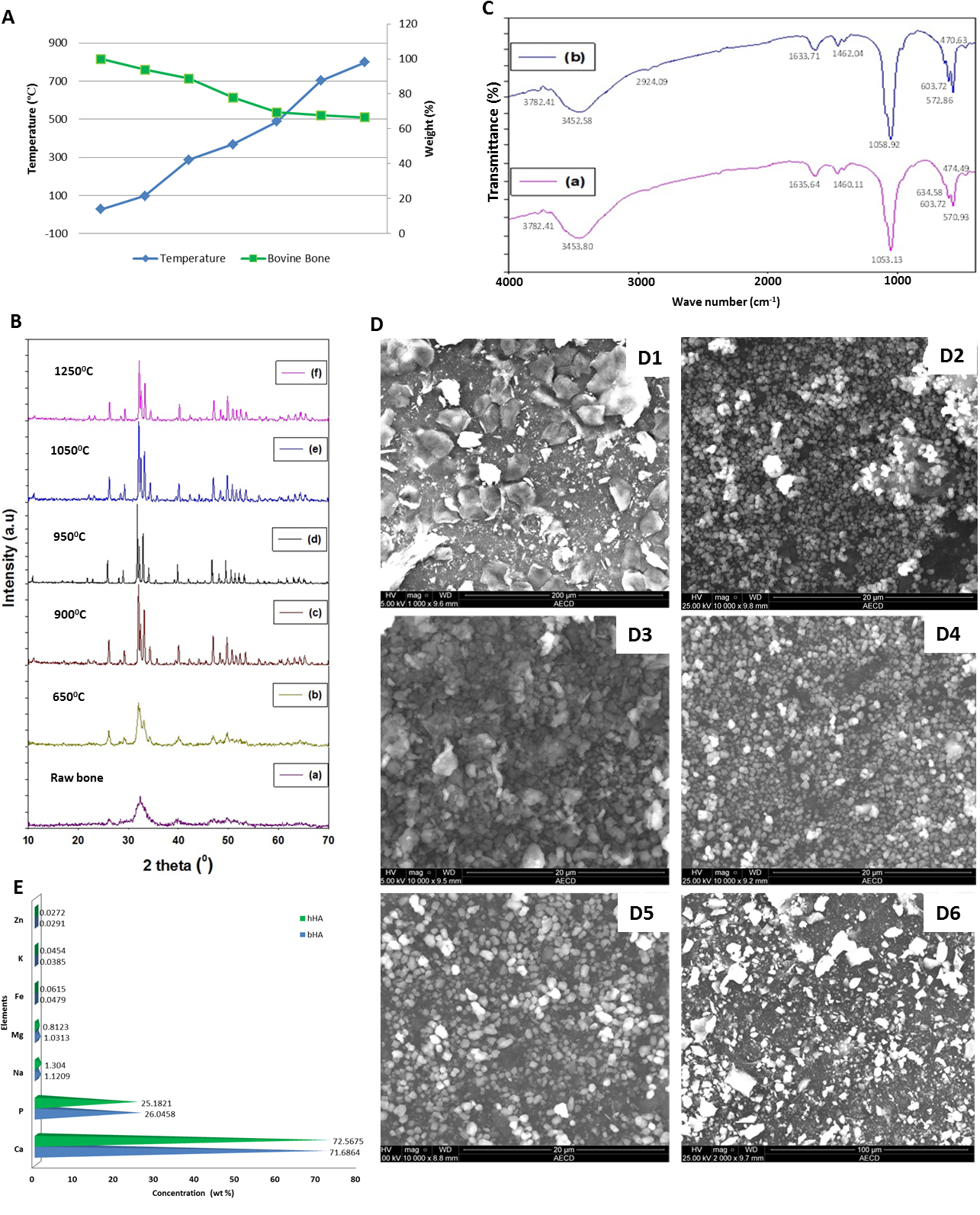


**Figure S1:** **Characterization of Ha nanopowder derived from bovine cortical bone.** (A) Thermo gravimetric analysis (TGA). (B) XRD analysis of raw bone (a) and Ha at various stages of annealing temperature - (b) 650°C, (c) 900°C, (d) 950°C, (e) 1050°C and (f) 1250°C.  C) FTIR results of - (a) Ha derived from bovine bone by annealing at 950°C and (b) commercial Ha as positive control. (D) SEM images of raw bone powder (D1), and Ha from bone sintered at various temperatures - (D2) 650°C, (D3) 900°C, (D4) 950°C, (D5) 1050°C and (D6) 1250°C.  (E) Elements of extracted Ha from XRF data.

**Characterization of Col1 isolated from rabbit skin**

Collagen was extracted from rabbit skin and the yield from ASC and PSC extraction methods were found to be 31% and 69%, respectively (figure S2A). The pH values of dialyzed samples of extracted collagen were stabilized in the range of 6.1–6.3, and no significant statistical differences between extractions (p>0.05) were observed (figure S2B). The thermal stability of Col1 was calculated from the thermal denaturation curve which was about 35.7°C (figure S2D). Two main collagen chains: α1 and α2 band were detected by SDS–PAGE. The Mw were of 130 and 115kDa, respectively. A third band was observed at 235kDa of β chain (figure S2E). The main absorption bands of collagen from FTIR were amide A (3299 cm^−1^), amide B (2950-2919 cm^−1^) and amide I (1632-1664 cm^−1^) with N-H stretching signature. Amide II (1500-1585 cm^−1^) and amide III (1200-1300 cm^−1^) were also observed (figure S2C). Glycine (40%) was detected as the most abundant amino acid in rabbit collagen beside hydroxyproline, alanine and proline (figure S2G). Three peaks were identified as α1, α2, and β chains from HPLC analysis. α1 chain was eluted first, followed by β and α2 chain (figure S2F).


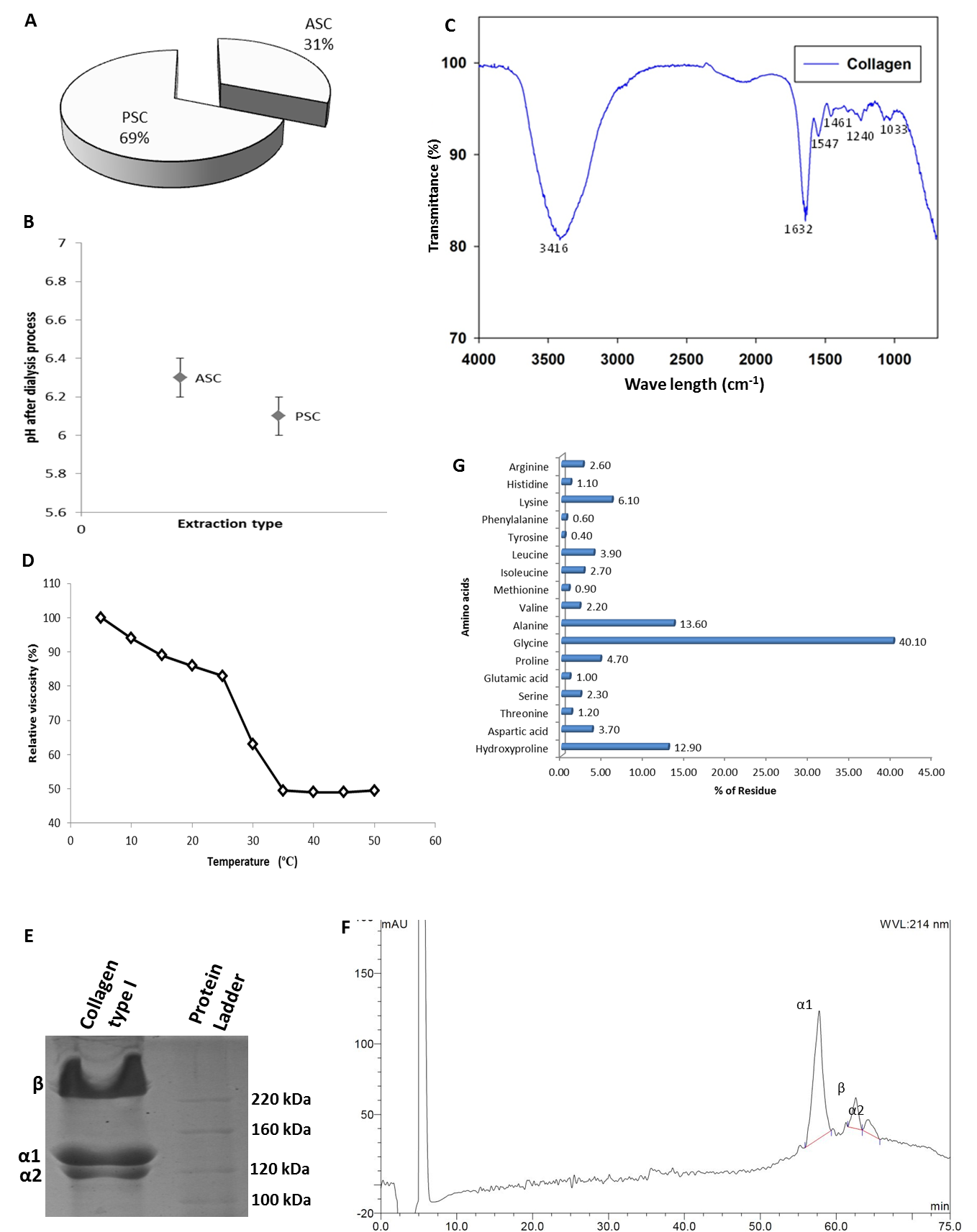


**Figure S2: Characterization of Col1 isolated from rabbit skin.** (A) Yield percentage of collagen using ASC and PSC methods. (B) pH value of extracted Col1 after 48 hrs of dialysis in DW at 20°C. (C) FTIR analysis showed the corresponding spectra of PSC extracted Col1 at 3416 cm^−1^ (Amide A) and at 1632 cm^−1^ (Amide 1). (D) Thermal denaturation curve of PSC collagen. (E) SDS-PAGE of collagen blot represents three distinct bands /Mw of alfa 1, alfa 2, and beta peptide. (F) Reversed-phase HPLC analysis of Col1. (G) Percentages of amino acid residues present in extracted Col1.
